# House-Walls

**Published:** 1866-04

**Authors:** 


### HOUSE-WALLS.

Wall-paper, like carpets, are the inventions of laziness and filth; they conceal dirt and noisomeness of every description. The almost milk-white floors and white plastered walls of the olden time have almost entirely disappeared, to the great detriment of family purity and personal health. It is greatly to be regretted that this is the case, to the extent that it is. White plastered walls can be kept clean for a number of years; the lime in them has the effect to purify them. Next to this is the painted wall, covered well with a suitable varnish; for it can be readily washed without injury, and is easily kept free of dust. In cases where walls must be papered, if for the first time, there are two important precautions: use no paper which has a green color, especially a "fuzzy green," which is composed of arsenic, and is capable of causing convulsions and fatal disease in a single night. Children have been taken extremely ill after playing a few hours in a small room covered with paper which had considerable green-colored patterns on it.

Care should be taken that the paste should be fresh, and put on equably and thin, and that any holes in the wall should be filled up with plaster. A tidy room in a certain dwelling was appropriated to lodgers. It was noticed, after a time, that as certainly as a person slept in that room a single night, severe sickness next day was the result. The authorities ordered an investigation, when it was found that a depression in the wall had been filled up by one of the workmen by gathering up a bucketful of pieces of paper, and some remnants of paste, to make them adhere. After a time, decomposition began to take place, giving out emanations of the most poisonous character; and for this reason, if any wall of plaster, or of wooden partition, is to be papered or re-papered, it should be thoroughly cleaned first, then made smooth; every particle of old paper should be removed.

The way in which the smallest amount of money can be made to go the farthest on a farm, morally and pecuniarily, is by investing it in lime and white lead. Filth, dirt, darkness, and untidiness, always and inevitably degrade those who dwell among them. Cleanliness purifies and elevates. If whitewash is used, it should be reäpplied twice a year to whatever is exposed to wind and weather; that which is, perhaps, the cheapest, most durable, and most generally available, is made thus: one ounce of white vitriol, that is, sulphate of zinc, and three ounces of common salt, to every four pounds of fresh lime, which is lime not fallen into any powder from exposure to the atmosphere, with water enough to make it sufficiently thin to be applied with a brush; this makes a durable out-door whitewash. When paint is used, two precautions are necessary: first, obtain a good article of white lead from a dealer whom you know to be honest. There is, perhaps, not one pound of pure white lead in a million that is sold for pure white lead, for there is a substance called barytes, which can be purchased by the ton for, perhaps, less than a cent a pound, which, when mixed with white lead, can not be distinguished until some time after it is spread, when it becomes dark; when it is remembered that white lead sells for ten times as much per pound, the temptation to adulterate is too strong for the honesty of any white lead manufacturer known to the writer. The proportion of this adulteration is from ten to ninety per cent.

Second. The preservative power of white paint depends, in considerable measure, on the time of year. If in hot weather, the water of the oil evaporates so quickly that the paint itself is not carried into the wood, and remains as a powder on the surface, and can be wiped off with the fingers. If in the inclement weather of winter, it is apt to be washed off by the rains before it has sufficiently dried. The autumn is best, when the ground is not likely to be dusty, and when the weather is long enough dry to allow the paint to get thoroughly dry itself.

Out-door wood-work should be painted once in every three years, and if done as just proposed, it not only preserves the building far beyond the cost of its application, but it gives an air of thrift, and life, and beauty, of which almost every reader has had personal experience. And in case of wishing to sell a farm thus kept painted and whitewashed, as to its fences and buildings, a better price can always be had, and from a better and more elevated class of purchasers.

